# circRNA-associated-ceRNA networks in patients with traumatic tracheal stenosis

**DOI:** 10.1038/s41598-025-24740-y

**Published:** 2025-11-19

**Authors:** Shujuan Qin, Wentao Li, Jinmei Wei, Yuhui Wei, Yinning Zhou, Pingping Huang, Sen Tan, Guangnan Liu

**Affiliations:** 1 Department of Respiratory and Critical Care Medicine, the Second Affiliated Hospital of Guangxi Medical University, Nanning, China; 2 Department of Pulmonary and Critical Care Medicine, Guigang City People’s Hospital, Guigang, China

**Keywords:** Tracheal stenosis, RNA, circular, RNA, messenger, Gene expression profiling, Sequence analysis, RNA, Functional genomics, Gene expression, Gene regulation, Sequencing

## Abstract

Traumatic tracheal stenosis (TTS) is one of the most commonly seen iatrogenic airway injuries caused by tracheal intubation or tracheotomy, which can harm the lives of patients in severe cases. Circular RNA (circRNA) is related to a variety of human diseases. However, the role that circRNAs are likely to play in traumatic tracheal stenosis is not known to a large extent till now. This research is designed to identify potential biomarkers and learn more about the mechanism of TTS through the establishment of a circRNA-associated ceRNA network. By carrying out RNA sequencing (RNA-seq), the expression profiles of circRNAs and mRNAs were explored from four TTS patients and four normal control cases. To investigate the differentially expressed circRNAs and mRNAs, this research performed both Gene Ontology (GO) enrichment and Kyoto Encyclopedia of Genes and Genomes (KEGG) pathway analyses. A circRNA-miRNA-mRNA network was predicted and established using bioinformatics tools including Mireap, Miranda, TargetScan, the circBase database, and miRTarBase. And then verified by utilizing RT-qPCR assays from six TTS patients and six normal control cases. In clinical tissue samples of the TTS group and the control group, 603 circRNAs and 1950 mRNAs were differentially expressed. According to GO and KEGG analyses, the differentially expressed circRNAs mainly participated in lysine degradation, platelet activation, and the VEGF/HIF-1 signaling pathway, whereas the differentially expressed mRNAs mainly participated in cytokine-cytokine receptor interaction, ECM-receptor interaction, neutrophil extracellular trap formation, and the PI3K-Akt signaling pathway. By utilizing 17 circRNAs, 7 miRNAs, and 9 mRNAs, a competing endogenous RNA (ceRNA) network was set up. Using RT-qPCR assay, It was confirmed that *S100A2* and circ_030284 were upregulated, while miR-1207-5p was downregulated in TTS patients compared to normal controls, consistent with the gene chip outcomes. The research presents a comprehensive circRNA-associated ceRNA network in patients with TTS, which provides novel insights into TTS’s underlying pathogenesis.

**Supplementary Information:**

The online version contains supplementary material available at 10.1038/s41598-025-24740-y.

## Introduction

Traumatic tracheal stenosis (TTS) represents one of the complications of tracheal intubation or tracheotomy^1^. Given the fact that critical care technology has been developed continuously, both tracheal intubation and tracheotomy have been applied to critically ill patients, and the incidence of traumatic airway stenosis has also increased^2–4^. The main cause of TTS is compression of the bronchial mucosa by the balloon used during tracheal intubation or tracheotomy, resulting in ischemia of the bronchial mucosa, proliferation of granulation tissue, and obstruction of the airway^5^. The histopathologic changes of TTS include inflammation, granulation tissue formation, and fibrosis, followed by narrowing of the tracheal cavity^6,7^. Endoscopic or open surgical approaches are the main treatment options for TTS^8^. Surgical resection and tracheal reconstruction were once the gold standard for treating TTS^7^. However, certain limitations still exist, such as poor tolerance of the procedure and the risk of postoperative complications, including anastomotic rupture or restenosis^9^. Despite serving as a minimally invasive procedure that has no external incision, endoscopy faces some inherent risks, such as mucosal laceration, laryngospasm, bleeding secondary to arterial injury, and restenosis^10,11^. Moreover, both medical interventional therapy and surgery were secondary damage that still caused tracheal stenosis^12,13^. No specific drug therapy for TTS exists now. Certain anti-inflammatory and anti-fibrosis drugs are in the early evaluation phases, though their moderate efficacy on TTS has been shown in animal experiments^14,15^. The management of tracheal stenosis is still controversial. Accordingly, airway stenosis should be treated by adopting a less invasive and more effective method, and it is significant to analyse the pathogenesis of traumatic tracheal stenosis.

As a new non-coding RNA class, circRNAs were first discovered around 40 years ago with a broad distribution in mice, fungi, plants, protozoa, human beings, etc^16,17^. It is formed by back-splicing through non-classical splicing. Different from traditional linear RNA, the expression of circRNA molecules cannot be easily degraded and is relatively stable since they are not influenced by RNA exonuclease and possess a closed-loop structure^18^. For a long time, it has been considered that circRNAs were the splicing errors’ products, until recently circRNAs’ properties were broadly and successfully analyzed through employing the new generation of RNA-seq and were also shown to contribute much to various biological processes^19^. Although the large-scale study of circRNA is not long, it has become one of the latest hotspots in the field of RNA research in recent years. CircRNAs involve frequent miRNA response elements that enable them to competitively bind to miRNAs (known as the sponge effect), leading to upregulation of downstream proteins^20^. Dysregulation of these signaling pathways is closely related to various human diseases, including immune diseases, cancers, viral diseases, and cardiovascular diseases^21–26^.In terms of tracheal stenosis, by comparing blood samples of tracheal stenosis samples with normal control samples, He et al.^27^ demonstrated that a total of 229 differentially expressed mRNA, 40 differentially expressed lncRNA, and 583 differentially expressed circRNA existed, which indicated that these genes are likely to be included in the pathogenesis of benign tracheal stenosis. Therefore, circRNA may become a new diagnostic marker or a therapeutic approach for certain diseases.

Our prior research has exhibited the miRNA and TTS correlation^28^, contributing to promoting TTS diagnosis and treatment. However, there are still many gaps in the research on the relationship between circRNA and TTS. This study explored the differential expression of circRNA genes between TTS samples and normal control samples, and in combination with previous studies, a circRNA-miRNA-mRNA regulatory network was also set up, which provided a reference for TTS’s biomarker mining and precision medicine.

## Materials and methods

### Patients

Between Jan. 2022 and Dec. 2022, this research recruited a total of 10 TTS patients on the first diagnosis at the Department of Respiratory and Critical Care Medicine in the Second Affiliated Hospital of Guangxi Medical University (GXMU). To be specific, four TTS patients and four normal controls were utilized for RNA sequencing, and 6 TTS patients and 6 normal controls were used for RT-qPCR. Each TTS patient was above 18 and was caused by tracheal intubation or tracheotomy with the diagnosis of computed tomography (CT), medical history, bronchoscope, and pathology. The research also excluded tracheal stenosis caused by tuberculosis, malignant and benign tumors, and other etiologies. A bronchoscopy was used to obtain TTS granulations. The research recruited 10 patients for the normal control group, all of whom came from those above 18 and experienced pulmonary lobectomies between Jan. 2019 and Dec. 2020 at the Department of Thoracic and Cardiovascular Surgery in the Second Affiliated Hospital of GXMU. From the resected bronchus, this research extracted normal bronchial mucosa, which was then verified through utilizing pathological analysis, implying that there was no tumor infiltration, inflammation, and fibrosis. Samples mentioned before were collected into an RNA preservation solution at 4 ℃ overnight and stored at -80 ℃ to conduct the following experiments. Prior to sample collection, informed consent was obtained from all participants. Each procedure was approved by the Ethics Association of the Second Affiliated Hospital of GXMU, in which the protocol reference was NO. **2022(KY-0043).** All those procedures were carried out in accordance with the institutional guidelines.

### RNA extraction, library construction, and sequencing

This research extracted the total RNA by employing the Trizol reagent kit (Invitrogen, Carlsbad, CA, USA) in accordance with the protocol of the manufacturer. RNA quality was evaluated through adopting the Agilent 2100 Bioanalyzer (Agilent Technologies, Palo Alto, CA, USA) and checked by utilizing the RNase free agarose gel electrophoresis. Upon the extraction of the total RNA, mRNAs and ncRNAs were both retained through the removal of rRNAs. The study fragmented the enriched mRNAs and ncRNAs into short fragments by employing the fragmentation buffer and next reverse transcribed them into cDNA by adopting random primers. By utilizing RNase H, DNA polymerase I, buffer, and dNTP (dUTP rather than dTTP), synthesis was carried out on second-strand cDNA. The cDNA fragments were then purified by utilizing the QiaQuick PCR extraction kit (Qiagen, Venlo, The Netherlands), end repaired, poly(A) added, and ligated to Illumina sequencing adapters. To digest the second-strand cDNA, this research next utilized UNG (Uracil-N-Glycosylase). The digested products were size selected through applying the agarose gel electrophoresis, underwent PCR amplification, and were sequenced by employing the Illumina HiSeq™ 4000 from the Gene Denovo Biotechnology Co. (Guangzhou, China).

### Identification of circRNA and characterization

The fastp^29^ (version 0.18.0) was utilized to filter raw reads obtained from the sequencing machines. Through employing Bowtie2^30^ (version 2.2.8), reads were mapped to ribosomeRNA (rRNA) database. A removal was carried out on the rRNA mapped reads. In the alignment and analysis, the research utilized the remaining reads further. By employing HISAT2^31^ (version 2.1.1), the rRNA with removed reads of all samples was next mapped to the reference genome. Upon the alignment with the reference genome, discarding was performed on the reads capable of being mapped to the genomes, and next the unmapped reads were collected to carry out circRNA identification. For the purpose of finding out unique anchor positions within the splice site, this study extracted 20mers from the two ends of the unmapped reads and then aligned them to the reference genome. To identify circRNAs, the circRNA splicing shown in the anchor reads that aligned in the reversed orientation (head-totail) was next subjected to find_circ^32^. Next, the research extended the anchor alignments and enabled the complete read alignments and breakpoints to be flanked by GU/AG splice sites. If it was supported by at least two unique back-spliced reads in a sample at least, then a candidate circRNA was called. Statistical analysis was performed to explore the type and chromosome distribution of the identified circRNAs.

### Identification of mRNA and characterization

The fastp^29^ (version 0.18.0) was used to filter the raw reads obtained from the sequencing machines. To map reads to ribosomeRNA (rRNA) databases, this study utilized the short reads alignment tool Bowtie2^30^ (version 2.2.8). A removal was next performed on rRNA mapped reads. In the assembly and gene abundance calculation, the remaining clean reads were employed further. A reference genome index was set up, and HISAT2^31^ and other parameters set were utilized as a default to map the paired-end clean reads to the reference genome. Through employing the StringTie v1.3.1^33^ within a reference-based method, the research assembled the mapped reads of all samples. As for all transcription regions, their expression abundance and variations were quantified by calculating an FPKM (fragment per kilobase of transcript every million mapped reads) value and a TPM (transcripts per kilobase of exon model every million mapped reads) value through the use of the RSEM software^34^.

### Analysis of differentially expressed genes and function enrichment analysis

Through utilizing the edgeR package^35^ (version 3.12.1) (http://www.r-project.org/), the circRNAs and mRNAs with a fold change ≥ 2 and a *P* value < 0.05 were identified by comparing the normal group or the TTS group as significantly differentially expressed circRNAs (DE circRNAs) and differentially expressed mRNAs( DE mRNAs). This research also carried out the Gene Ontology (GO) by DAVID Tool (http://david.ncifcrf.gov/gene2gene.jsp), consisted of annotations of the concerned biological processes (BP), cellular components (CC) and molecular functions (MF). And the Kyoto Encyclopedia of Genes and Genomes (KEGG) (https://www.kegg.jp/) pathway analyses and therefore predicted the biological functions of DE circRNAs and DE mRNA^36,37^. Heatmap, volcano, GO and KEGG plots are visualized using the edgeR package^35^ (version 3.12.1).

### Integrated analysis of DE circRNAs-DE miRNAs-DE mRNAs

The StarBase (version 2.0)^38^ can be utilized to predict the target connection between circRNAs in the circBase database and miRNAs. The targets of the novel circRNAs were predicted by employing the three software, which were Mireap(https://sourceforge.net/projects/mireap/), Miranda (version 3.3a) (https://miranda-ng.org/) and TargetScan (version 7.0)^39^. This research employed miRTarBase (version 6.1)^40^ to predict mRNAs targeted by miRNA sponge, therefore finally realizing the prediction of mRNAs that interacted with circRNAs and miRNAs. In combination with the differentially expressed miRNAs data (DE miRNAs) that were analyzed in our previous study^28^ (Table S1), the miRNAs-DE mRNAs network was constructed. Finally, Cytoscape was used to visualize the resulting circRNAs- miRNAs-mRNAs relationship^41^.

### Verification of hub circRNAs, miRNAs, and mRNA by qRT-PCR

Through integrated bioinformatics analysis(Mireap, MIRanda, TargetScan, miRTarBase and circBase), we established a competing endogenous RNA network. Given that inflammation and fibrosis represent the predominant histopathological manifestations in TTS, we prioritized the hsa_circ_030284/hsa-miR-1207-5p/*S100A2* axis for experimental validation via qRT-PCR.Through employing the reverse transcriptase (Takara, China) in accordance with the protocol of the manufacturer, the total RNAs of 6 of TTS tissues and 6 of normal control tissues extracted by utilizing the TRIzol reagent (Takara, China) were then reversely transcribed. The qRT-PCR was then performed by applying the QuantStudioTM 5 Real-Time PCR System (Agilent Technologies) by utilizing the SYBR Green PCR Kit (Takara) in accordance with the manufacturer’s protocol. The PCR amplification protocol was conducted under the following thermal cycling conditions: initial denaturation at 95 °C for 3 min, followed by 40 cycles of denaturation at 95 °C for 10 s, and annealing/extension at 60 °C for 30 s.The research leveraged the Sangon Biotech (Shanghai, China) to perform chemical synthesis of primers that were utilized for qRT- PCR. Sequences of those primers were exhibited in Table 1. Normalization was performed using GAPDH for both circRNA and mRNA quantification, while U6 small nuclear RNA was employed as the endogenous reference for miRNA detection. Relative expression levels were calculated using the comparative threshold cycle (2-ΔΔCt) method.

**Table 1:** The primers’ sequence for qRT-PCR.

Gene	Primer sequence
U6-Foward	CTCGCTTCGGCAGCACA
U6-Reverse	AACGCTTCACGAATTTGCGT
GAPDH- Foward	AGAACATCATCCCTGCCTCTACTGG
GAPDH- Reverse	CGCCTGCTTCACCACCTTCTTG
has_miR-1207-5p-Forward	AATAATGGCAGGGAGGCTGGGAGG
has_circ_030284-Forward	TCAAAACCTGTGGCTAGCAC
has_circ_030284-Reverse	CTTCATCTGCATGCACATGTAC
*S100A2*-Forward	CCCACGACACCTCCCACTTC
*S100A2*-Reverse	CCTCTTGCCATTCCCACTCATTC

### Statistical analysis

Through the respective use of SPSS 26.0 and GraphPad Prism 8, the research realized data statistical processing and graph creation. The data normality was investigated, the Student’s t-test was utilized for data that conformed to the normal distribution, and the Mann–Whitney U test was used for data not matching with the normal distribution. The research defined the statistical significance as *P* < 0.05.

## Results

### Overview of circRNA identification

**Fig. 1:**
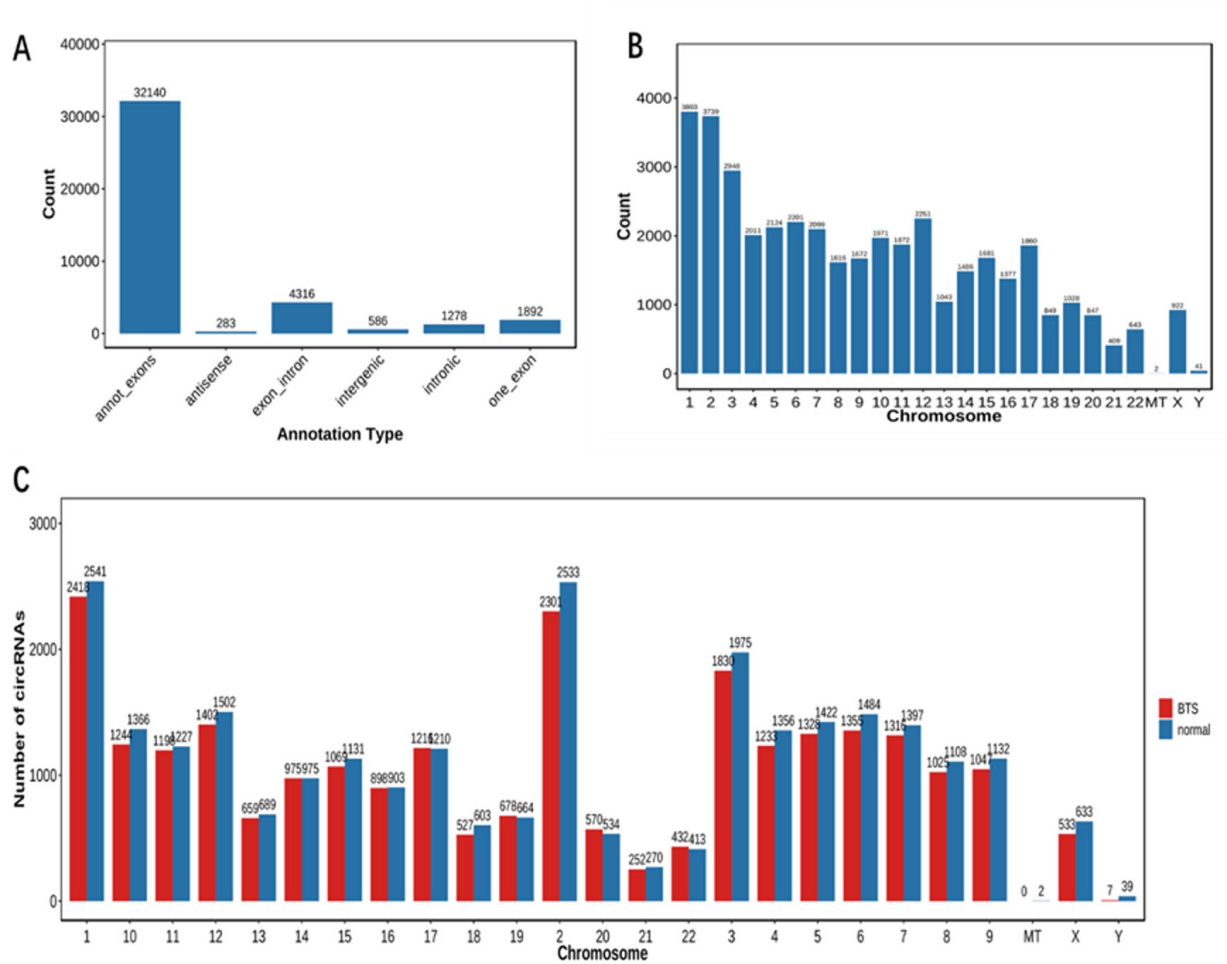
(**A**) Distribution of cirRNA types; (**B**) map of the chromosomal distribution of circular RNA; (**C**) distribution of circular RNA chromosome numbers in each group.

The research recruited four TTS (BTS-1, BTS-2, BTS-3, and BTS-4) and four normal control patients (normal-1, normal-2, normal-3, and normal-4), respectively, to conduct small RNA sequencing. Table 2 shows this general information. The serial high-throughput sequencing identified 40,495 circRNAs in total, along with 13,374 existing circRNAs and 27,121 novel circRNAs, which contained 10,549 existing circRNAs, 14,964 novel circRNAs of the TTS group and 10,009 existing circRNAs and 17,100 novel circRNAs of the normal group (Table 3). The sequencing in the exon region gathered more than 80% reads, and most cirRNA types were annot_exons (32140) and exon_intron (4316) (Fig. 1A). These circRNA distributions in chromosomes were illustrated in Fig. 1B,C.

**Table 2:** General information of patients.

Patients	Sex	Age		Diagnose
BTS_1	Male		56	Post-tracheotomy stenosis
BTS_2	Male		61	Tracheal stenosis after intubation
BTS_3	Female		45	Post-tracheotomy stenosis
BTS_4	Female		62	Tracheal stenosis after intubation
Control_1	Male		59	Lung squamous cell carcinoma
Control_2	Male		65	Lung adenocarcinoma
Control_3	Female		48	Lung adenocarcinoma
Control_4	Female		64	Lung squamous cell carcinoma

**Table 3:** The summary of sequencing data.

group	total_circRNA	exist_circRNA	novel_circRNA
ALL	40,495	13,374	27,121
BTS	25,513	10,549	14,964
Normal	27,109	10,009	17,100

### Differentially expressed circRNAs

**Fig. 2:**
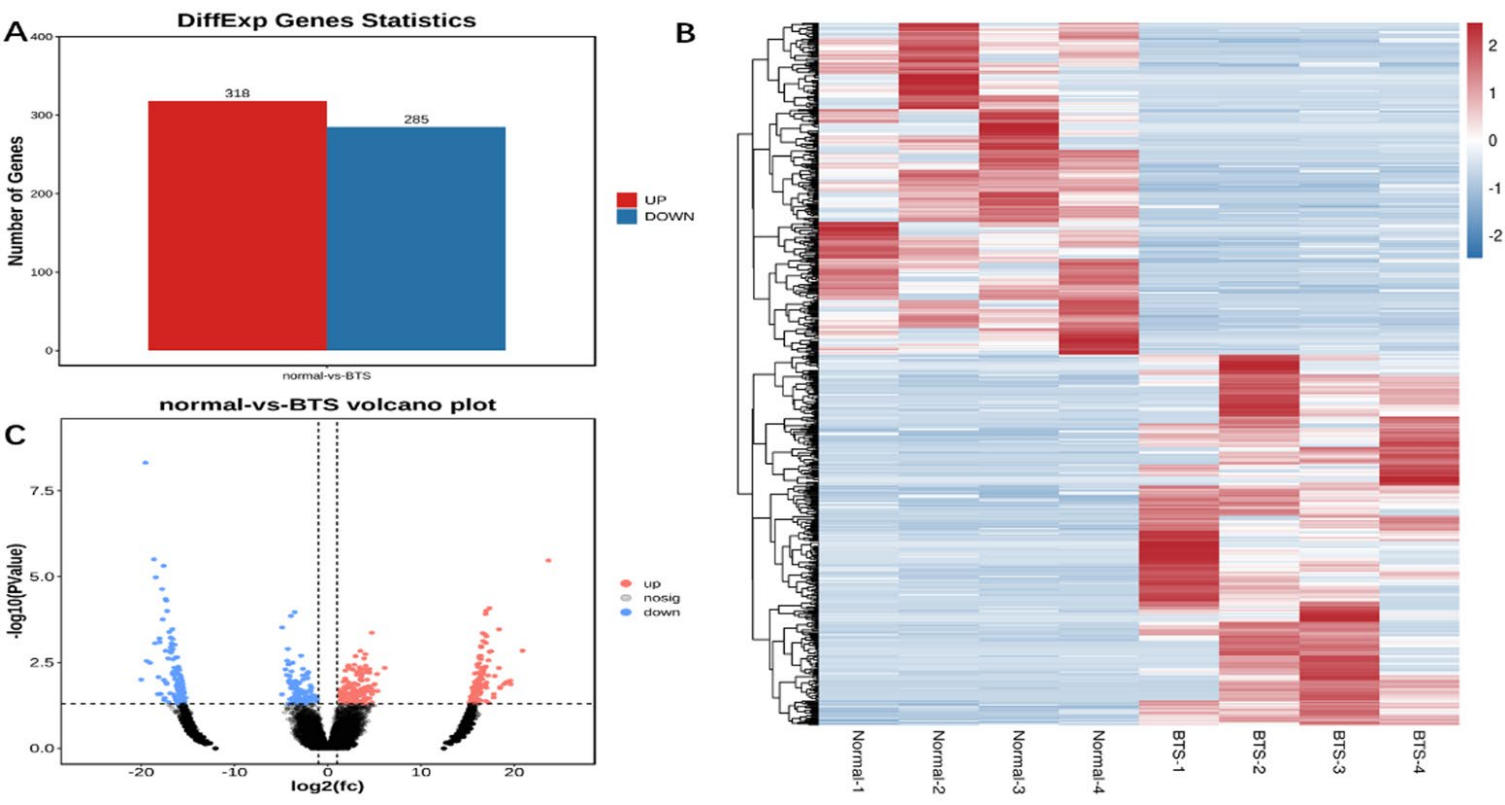
(**A**) Statistical map of differential circRNA; (**B**) circRNA difference analysis heat map; (**C**) circRNA difference analysis volcano map.

Through leveraging the|log2 fold change| >1 and corrected p-value < 0.05 as the cut-off, the research further assessed the significant differential expression of circRNAs from the TTS and normal control groups. Besides, we also collected a total of 603 circRNAs that had significant differential expression (Fig. 2A and Table S2). Among these circRNAs, which were named DEcircRNAs, 318 were up-regulated, and 285 were down-regulated. Figure 2B,C presented the hierarchical clustering and volcanic maps and heatmap.

### Enrichment analysis of differentially expressed circRNA

**Fig. 3:**
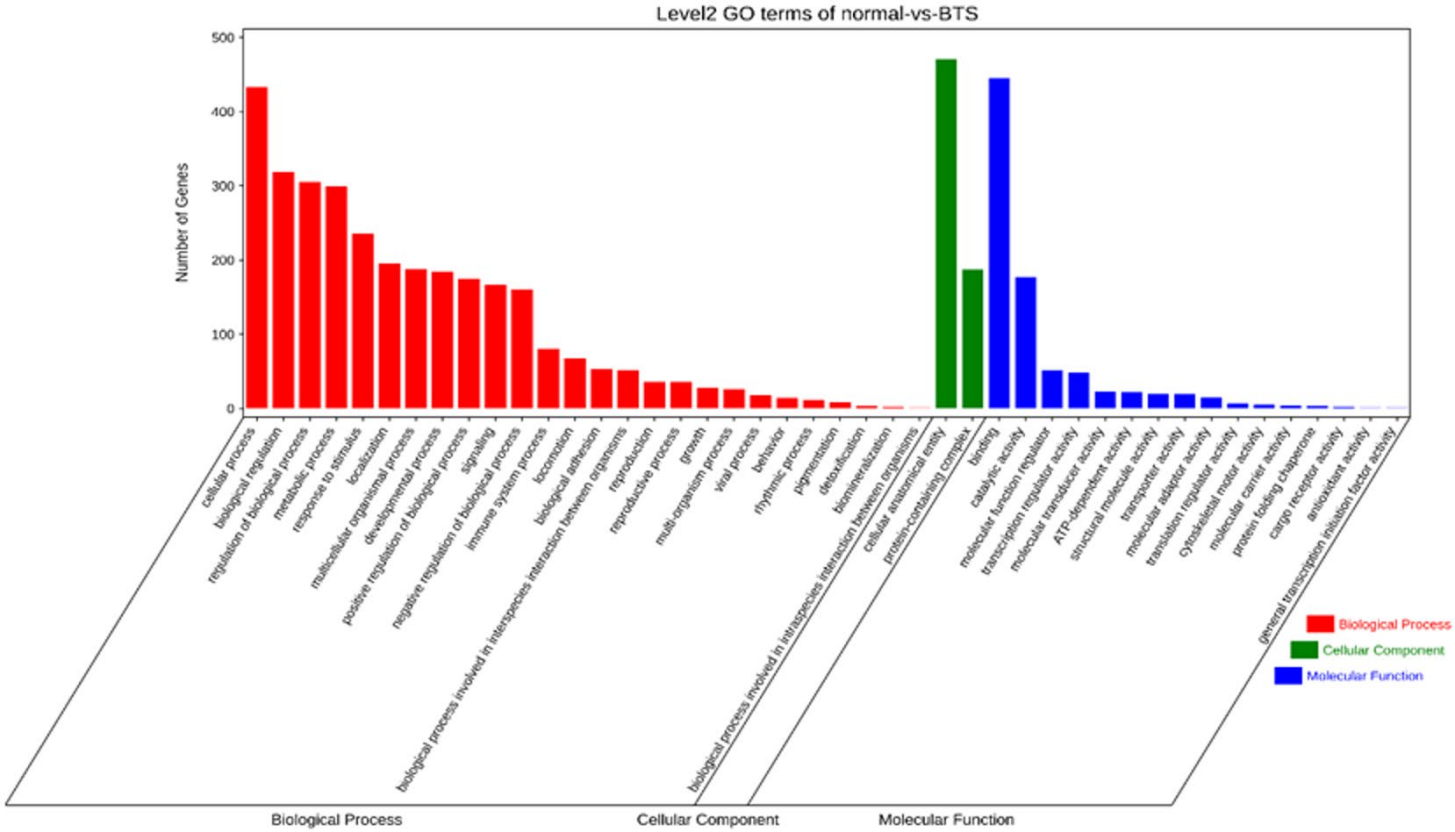
GO gene function classification of target genes of circRNAs with differential expression.

**Fig. 4:**
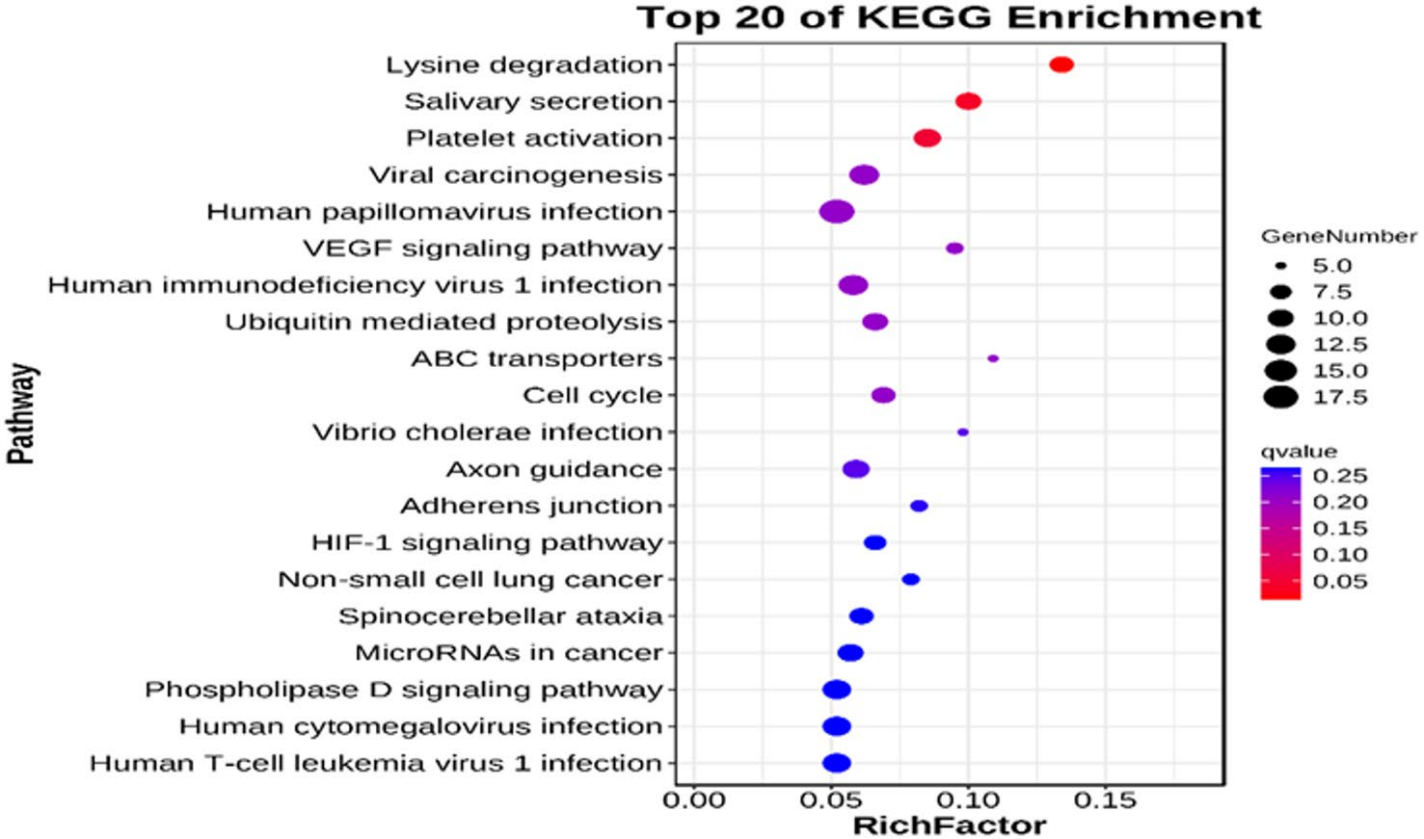
Bubble diagram of the signal pathway linked to the KEGG enrichment of candidate genes.

To elucidate the functional implications of differentially expressed circRNA, we performed GO analysis and KEGG pathway analysis. As can be found in the enrichment analysis of differentially expressed circRNAs, those differentially expressed circRNAs had a correlation with biological processes, including cellular process, regulation of the biological process, metabolic process, biological regulation, response to stimulus, and the involved molecular functions, such as binding, ATP-dependent activity, molecular function regulator, catalytic activity, and transcription regulator activity (Fig. 3 and Table S3). Differentially expressed circRNAs were contained within certain pathways, including platelet activation, lysine degradation, the VEGF-signaling pathway, salivary secretion, and the HIF-1 signaling pathway (Fig. 4 and Table S4).

### Identification of differentially expressed mRNAs

**Fig. 5:**
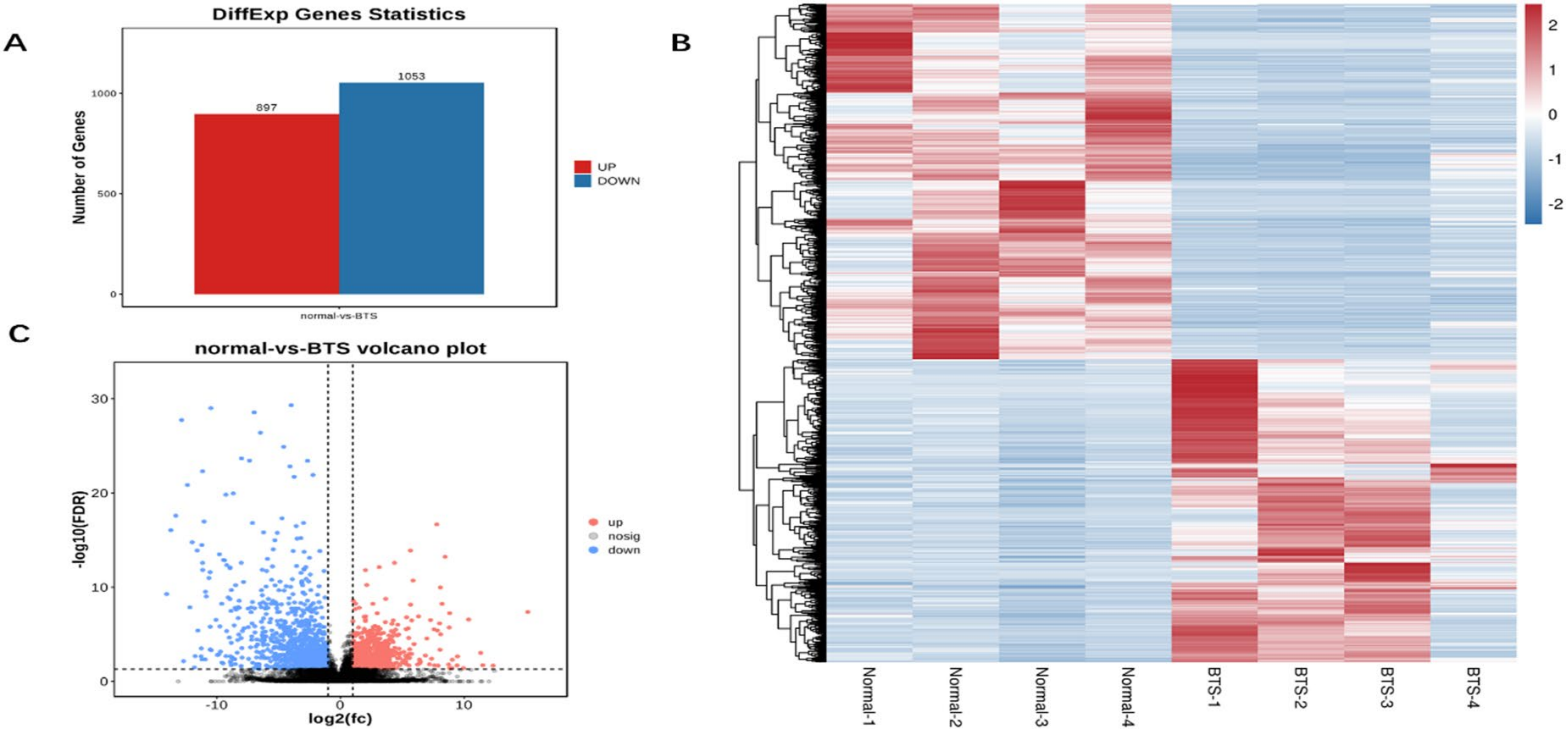
(**A**) Statistical map of differential mRNA; (**B**) Heatmap and clustering analysis of DE mRNAs; (**C**) Volcano plot of DE mRNAs in BTS tissues compared with controls.

The changes in mRNA expression were analysed under the same conditions as well. In the cluster heatmap, differential expression patterns of mRNAs in BTS tissues were shown in comparison with the controls. 20,323 mRNAs in total were found, with 1,950 mRNAs being differentially expressed between both groups, and up-regulation occurred in 897 mRNAs and down-regulation emerged in 1,053 mRNAs, respectively (Fig. 5A–C and Table S5).

### Enrichment analysis of differentially expressed mRNA

**Fig. 6:**
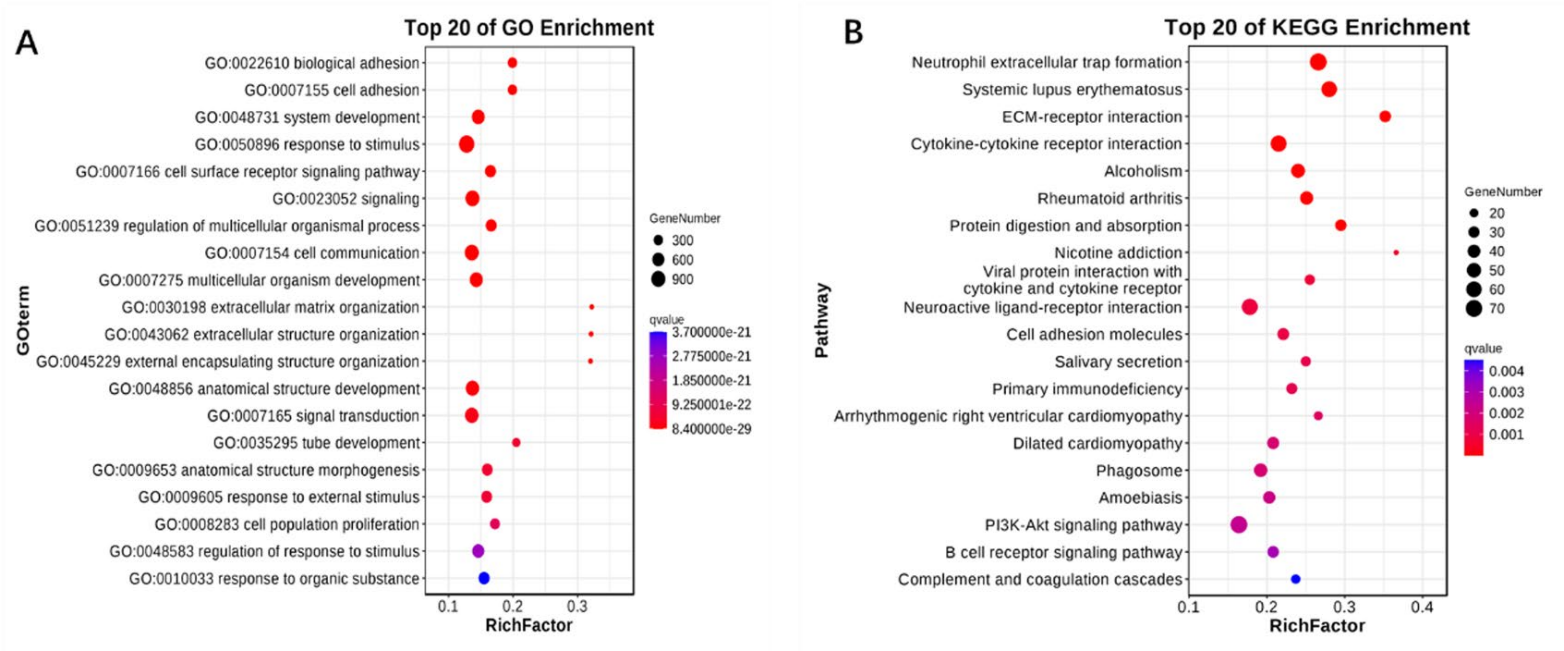
Enrichment analysis of differentially expressed mRNA. (**A**) and (**B**), respectively, exhibit the top 20 GO biological process terms and KEGG pathway terms of DE mRNAs.

The differentially expressed mRNAs underwent GO enrichment and KEGG pathway analyses. According to the GO enrichment analysis, some biological processes presented high enrichment of the differentially expressed mRNAs, including signaling, response to stimulus, cell communication, extracellular matrix organization, and cell surface receptor signaling pathway (Fig. 6A and Table S6). Besides, as can be found in the KEGG pathway analysis, a variety of mRNAs are mainly included within ECM-receptor interaction, neutrophil extracellular trap formation, the PI3K-Akt signaling pathway, and cytokine-cytokine receptor interaction (Fig. 6B and Table S7). Those data suggested that activation of the cellular inflammatory response occurred within TTS.

### Construction and analysis of the circRNA-miRNA-mRNA networks

**Fig. 7:**
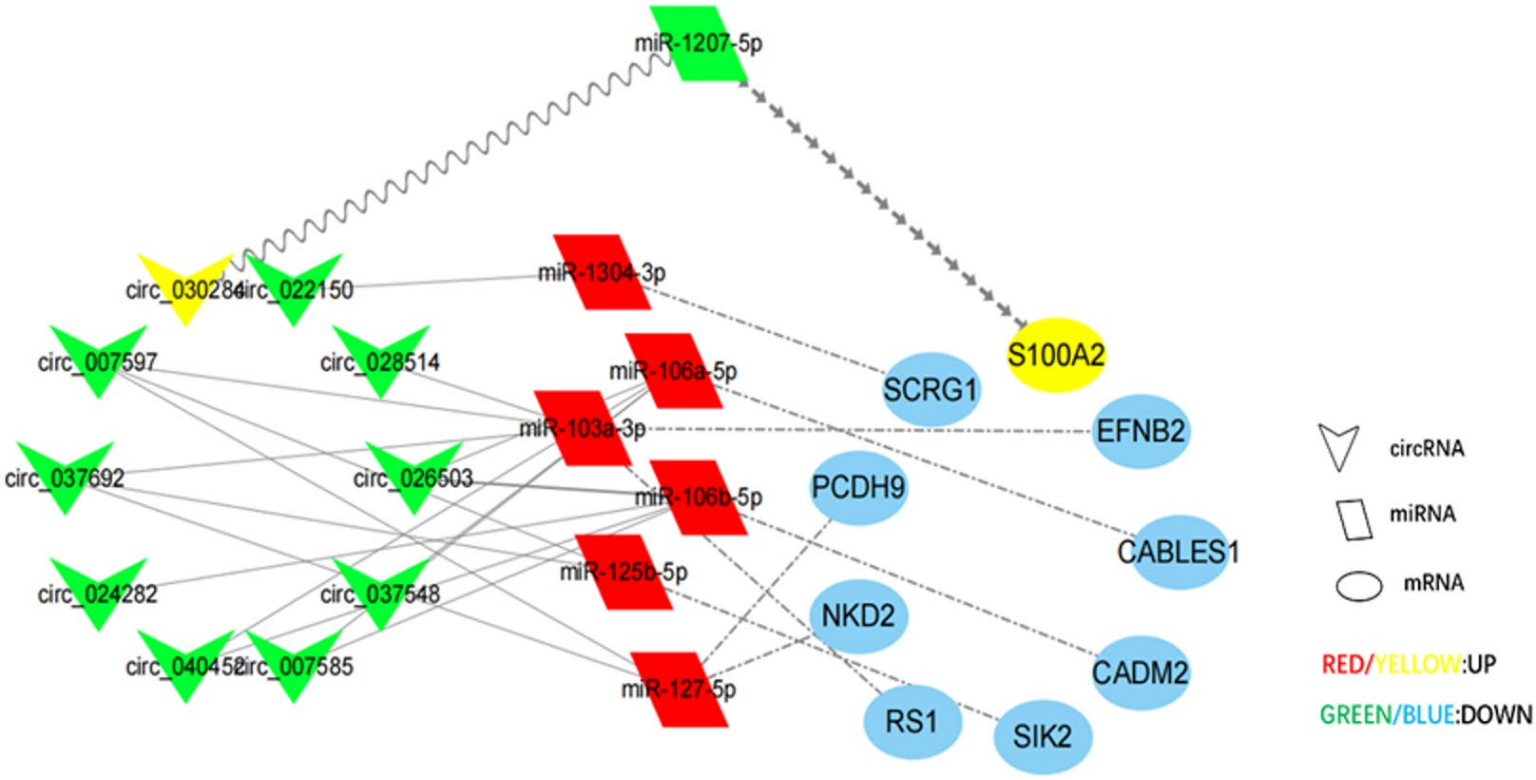
CircRNA-miRNA-mRNA ceRNA Network.

Given the ceRNA hypothesis, circRNAs act as miRNA sponges to sequester miRNAs, which indirectly upregulates the expression of mRNA target genes, this research explored the coordinated networks in those differentially expressed RNAs. For 603 DE circRNAs, 1950 DE mRNAs, and DE miRNAs in our prior research^28^, we set up 7 circRNA-miRNA-mRNA pathways, which contained 7 miRNAs, 9 mRNAs and 18 circRNAs (Fig. 7 and Table S8).

### The result of qRT-PCR

**Fig. 8:**
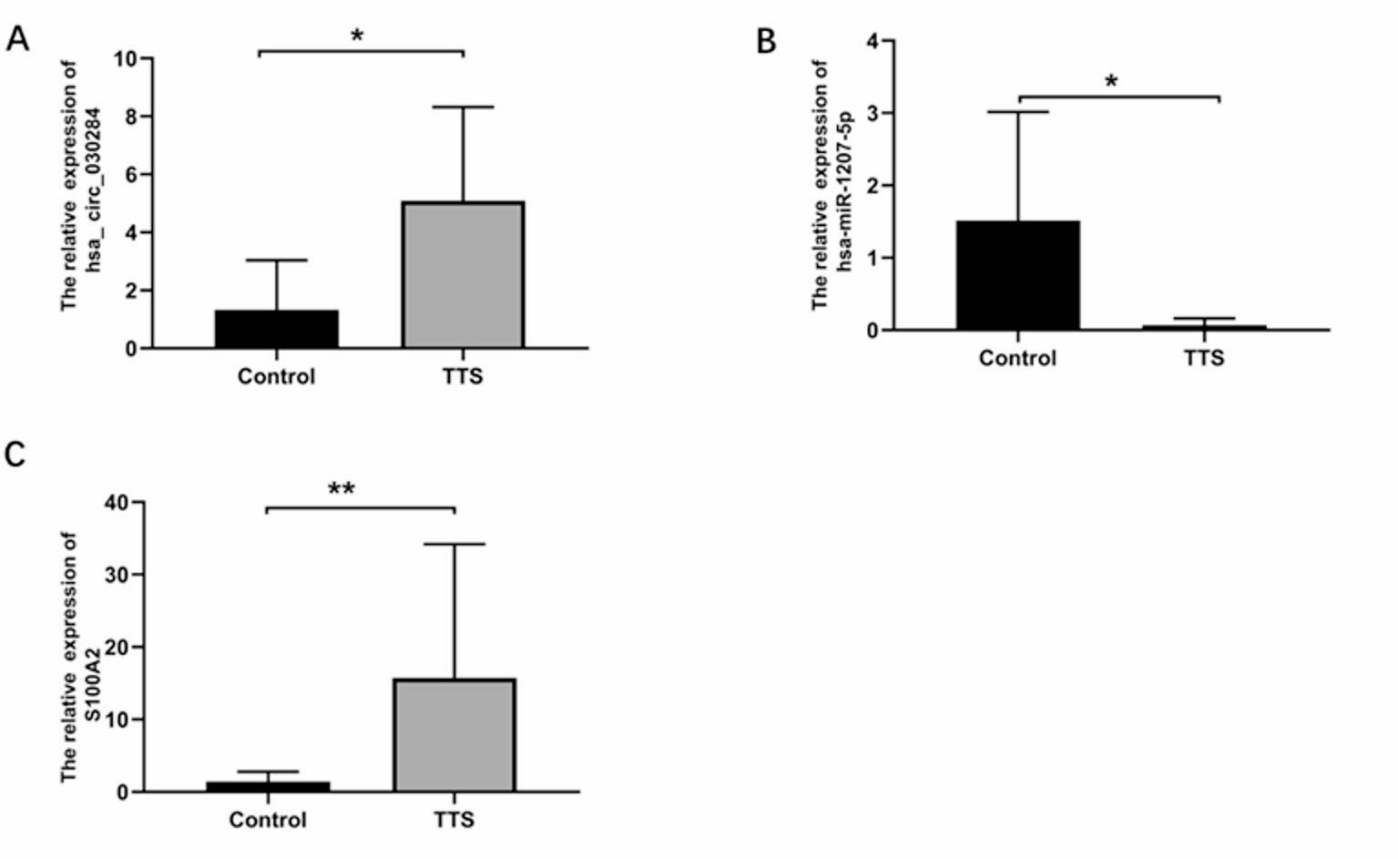
Verification of the hsa_circ_030284/hsa-miR-1207-5p/*S100A2* regulatory axis. (**A**–**C**) The expression levels of hsa_circ_030284 (**A**), hsa-miR-1207-5p (**B**), and *S100A2* mRNA (**C**) were determined by RT-qPCR.

To further analyse the correlations between relative target RNAs and TTS, hub circRNAs and mRNAs that had high expression levels were chosen from the sequencing data. Besides, for investigating the correlations between the target circRNAs, target miRNAs and target mRNAs in the ceRNA sub-network, their expression levels in TTS tissues were further explored through the use of qRT-PCR (Fig. 8A–C and Table S9).

Elevated expression of *S100A2* and circ_030284 was observed in TTS patients compared to normal controls, whereas a reduced expression level of miR-1207-5p was detected. In addition, by conducting the mRNA–circRNA correlation analysis, it was judged that *S100A2* expressions had a positive relationship with hsa_circ_030284 expression. Through performing miRNA-mRNA correlation analysis in the tracheal stenosis tissues, it was identified that hsa-miR-1207-5p had a negative correlation with the target genes *S100A2*. What’s more, hsa_circR_030284/hsa-miR-1207-5p/*S100A2* expression conformed to ceRNA mechanism characteristics.

## Discussions

Tracheotomy and intubation induced tracheal stenoses are both forms of traumatic airway stenosis with identical pathogenesis and pathological characteristics. Pathological change of TTS is primarily characterized by submucosal hypertrophy resulting from the proliferation of fibroblasts and thickened collagen fibers and the increase of the intensive infiltration of inflammatory cells^42^. Inflammation, hypoxia-induced damage, abnormal repair, and fibrosis can cause both the proliferation of scar tissue in the trachea and the formation of fibrous scars^43^. Therefore, the essence of TTS is aberrant airway fibrosis. Although the current TTS treatment primarily involves surgery, drug therapy, and interventional therapy, TTS management still serves as a challenge in respiratory interventional therapy^14,44^. Hence, TTS pathogenesis needs to be analysed and efficient approaches should be applied to control its development. Through leveraging transcriptome sequencing, the expression of TTS and control transcripts in this research was collected quickly. The papers on less whole transcriptome sequencing associated with this disease are relatively fewer till now. In this present study, the innovation lies in analyzing possible therapeutic methods for that disease and jointly exploring expression profiles of circRNAs, miRNAs and mRNAs in TTS patients through utilizing the tracheal tissue samples.

As a newly described non-coding RNA type, circRNAs are characterized by conservation, biological stability, and tissue specificity. Firstly acting as sponges for RNA-binding proteins (RBPs), circRNAs are included in protein translation and can regulate transcription, thereby providing circRNAs with higher regulator potential for developing fibrotic diseases^45–47^. Through a comparison of tracheal stenosis tissues and normal control tissues, the research identified a total of 1950 differentially expressed mRNA and 603 differentially expressed circRNA, which implied that those genes are likely to be included in TTS pathogenesis. Both GO and KEGG analyses were carried out on those differentially expressed mRNAs and circRNAs. According to the functional enrichment analysis, significant enrichment emerged in the differential mRNAs within biological processes, such as signaling, response to stimulus, cell communication, cell surface receptor signaling pathway, and extracellular matrix organization.

In the KEGG pathway analysis, it can be found that a variety of DE mRNAs are mainly included within ECM-receptor interaction, neutrophil extracellular trap formation, the PI3K-Akt signaling pathway, and cytokine-cytokine receptor interaction. In addition, it can be found from the functional enrichment analysis that significant enrichment occurred in the differentially expressed circRNAs within biological processes, including biological regulation, cellular process, metabolic process, regulation of biological process, response to stimulus, and the included molecular functions, like molecular function regulator, binding, transcription regulator activity, catalytic activity, and ATP-dependent activity. KEGG pathway analysis indicated that DE circRNAs were included within certain pathways, including the VEGF-signaling pathway, lysine degradation, platelet activation, salivary secretion, and HIF-1 signaling pathway.

TTS development is speculated to be associated with the activation of inflammation and fibrosis^43^. The pressure of the balloon on the tracheal mucosa after tracheal intubation causes mucosal ischemia and hypoxia, resulting in inflammatory response and fibrosis^5^. The signaling between immune cells and fibroblasts plays a necessary role in TTS fibrosis and scarring. Due to the injury of tracheal mucosa, an inflammatory response is triggered, which leads to up-regulated cytokines, including tumor necrosis factor a (TNF-α), IL-1α, fibroblast growth factor (FGF), IL-6, and platelet-derived growth factor (PGDF). Subsequently, those cytokines drive the fibroblast activation and differentiation into a myofibroblast phenotype^43^. It has been reported that after the operation on Sprague–Dawley (SD) rats, the tissue wound was along with much inflammatory cell infiltration and increasing fibroblasts in the thickened submucosa with collagen deposition^42^. In addition, certain research exhibited that platelets attended physiological coagulation and facilitated both inflammation development and wound healing^48,49^.

There is the occurrence of platelet activation in various inflammatory and fibrotic diseases, such as asthma, IPF, and hepatic fibrosis^50–52^. In the early stage of tracheal tissue injury, hemostasis is triggered by the subendothelial extracellular matrix (ECM) exposure and then platelets are activated. At the same time, damaged epithelial or endothelial cells produce and release excess inflammatory mediators that induce subsequent leukocyte entry through the ruptured basement membrane^43^. The neutrophil activation and recruitment are promoted by activated platelets. Through transforming growth factor-β (TGF-β), and secreting proinflammatory cytokines IL-6 and TNF-a and profibrotic cytokine PDGF, neutrophils promote inflammation development and recruit other inflammatory cells to the wound^43^. PDGF, IL-1 and TGF-β promote myofibroblast aggregation. Myofibroblasts are the main source of ECM components and therefore are highlighted as the main effector cells in fibrosis understanding myofibroblast origin in the fibrotic lung. Fibrous scar formation is a compensatory repair after injury.Chronic hypoxia is actively included in fibrosis pathogenesis. The research enriched the fibrotic signaling pathways, which included the HIF-1 signaling pathway, the PI3K-Akt signaling pathway, and the VEGF-signaling pathway, as well. At the cellular level, oxygen perception and response included the HIF (hypoxia-inducible factor) and its regulator pVHL (VonHippel-Lindau tumor suppressor protein). Under hypoxia, pVHL does not recognize the HIF-1α subunit, which activates and enters the nucleus, where it interacts with the co-factors CBP/p300 and Pol II complexes and binds to hypoxia response element (HRE) for activating the target genes’ transcription. It involves energy metabolism, angiogenesis, cell differentiation, cell proliferation and apoptosis, invasion and metastasis, and treatment resistance^53^.Airway stenosis has a close correlation with mechanical compression. In the case of balloon compression, the HIF-1 signaling pathway is activated since the tracheal mucosa is in a state of ischemia and hypoxia^54^. The activation of HIF-1 signaling regulates many important profibrotic enzymes and proteins that can stimulate cells to secrete extracellular matrix^43^.The primary biological function lies in regulating fibroblast activity and promoting angiogenesis. Additional studies have found that the HIF-1α /VEGF signaling pathway contributes much to acute lung injury pathogenesis^55^. Hypoxia-induced HIF-1α synthesis promotes VEGF and TGF-β overexpression, and the excessive activation and proliferation of fibroblasts leads to airway scar formation^56^.The previous study of our research group identified thickened submucosa of tracheal stenosis, observed neovascularization^42^, and highly expressed VEGF in the tissue of TTS. After the erythromycin intervention, the VEGF expression was significantly down-regulated, the proliferation of tracheal epithelial tissue was significantly improved, and the tracheal stenosis degree was also significantly reduced^57^. All these suggested that the VEGR signaling pathway is related to the formation of traumatic tracheal stenosis. However, it is necessary to further confirm whether HIF-1/VEGF signaling and other signaling pathways mentioned contribute to TTS pathogenesis, but it provides a direction for studying the pathogenesis and treatment of this disease.

According to the competitive endogenous RNA (ceRNA) hypothesis, there are miRNA recognition elements in circRNA that can both adsorb and inhibit miRNA function. The RNA interactions were taken as the basis to set up a ceRNA network that included a total of 17 circRNAs, 7 miRNAs, and 9 mRNAs. By employing qRT-PCR validation, we identified one of the ceRNA networks from the network: hsa_circ_030284/has-miR-1207-5p*/S100A2.*The expression levels of *S100A2* and hsa_circ_030284 displayed higher expression levels in patients that had TTS than those in normal controls. Moreover, down-regulation was seen in miR-1207-5p expression levels within patients that had TTS. In the axis here, hsa_circ_030284 bound to has-miR-1207-5p and next released the inhibitory effect of has-miR-1207-5p on *S100A2*. S100A2 serves as an S100 protein family member, contributing to human physiology and pathology through regulating downstream target proteins’ activity^58^. In the S100 protein family, there are more than 20 members that have unique structural features, which involve an N-terminal (N-) and a C-terminal (C-) cluster, and two EF-hand calcium-binding structural domains separated by a hinge region^59^. Compared to the non-regular N-terminal EF hand, the affinity of the C-terminal EF hand for calcium ions is 100 times greater. There are differences in the C-terminal regions of the different S100 proteins, which may provide selective binding sites for the specific target proteins^60^.

S100A2 is linked to cell proliferation and differentiation, and studies have found that the expression of S100A2 is enhanced after lung epithelial cells are long-term exposed to low-dose nickel-containing nanoparticles, indicating the inclusion of S100A2 in pulmonary inflammatory diseases’ occurrence^61^. Another study found increased salivary S100A2 levels in patients who had long-term uncontrolled asthma, suggesting that airway inflammatory diseases are also associated with *S100A2*-mediated pathogenesis^62^. Huang et al.^63^ found the increased expression of S100A2 in patients who had pulmonary fibrosis. Furthermore, this research implied that the *S100A2* knockdown drives epithelial-mesenchymal transition (EMT) within lung epithelial cells by antagonizing the Wnt/β-Catenin signaling pathway, and through inhibiting downregulated kinases, which were p-GSK-3β and β-Catenin, suggesting that S100A2 is a promising potential target for further understanding the pathogenesis of pulmonary fibrosis and development of therapeutic strategies. Therefore, S100A2 deserves further investigation.

Regarding hsa-miR-1207-5p, miR-1207-5p was primarily characterized as an EMT negative regulator through controlling the expression of certain genes, such as *SMAD 2*, *SMAD 3*, *SMAD 7*, *CLASP 1*, *ZEB 1* and *SNAIL 1*. Apart from the role it plays in EMT, miR-1207-5p also contributes much to shaping the inflammatory microenvironment. From that perspective, it has been reported that *CSF1* (colony-stimulating factor 1) is one of its direct targets^65^. The EMT process favors fibrotic events. According to Giorgio et al.^66^, the hsa-miR-1207-5p is likely to cause *CSF-1* dysregulation through interacting with the SARS-CoV-2 viral genome, thereby enhancing inflammatory response and promoting EMT, leading to the occurrence of pulmonary fibrosis. hsa_circ_030284 has not been reported so far.

The research showed increased circ_030284 expression, decreased miR-1207-5p expression, and increased *S100A2* expression in the tracheal granulation tissue of patients that had TS, conforming to the sequencing results. This suggests that hsa_circ_030284/hsa-miR-1207-5p/*S100A2* may be included within TTS occurrence and development, which should be analysed further. By leveraging qRT-PCR, it was verified that circ_030284 indirectly regulated *S100A2* expression through sponging miR-1207-5p, which may be involved in the occurrence and development of traumatic tracheal stenosis. Additionally, this study has certain limitations. Despite the validation of circRNA-miRNA-mRNA in the ceRNA network through utilizing qRT-PCR and the dataset, the role of the ceRNA axis in TTS should be verified through further experiments, including dual luciferase reporter assay, overexpression and inhibition assay, and western blotting.

## Conclusions

This research employed small RNA sequencing to detect circRNAs and mRNAs of granulation tissues from the TTS patients. We utilize bioinformatics methods to analyze the main functions of key genes, laying a basis to further explore TTS treatment. We also constructed a circRNA-associated ceRNA regulatory network, which offered a novel direction to analyse the TTS pathogenesis further.

## Supplementary Information

Below is the link to the electronic supplementary material.

Supplementary Material 1

Supplementary Material 2

Supplementary Material 3

Supplementary Material 4

Supplementary Material 5

Supplementary Material 6

Supplementary Material 7

Supplementary Material 8

## Data Availability

The sequencing data involved in this study have been publicly available and stored in the National Center for Biotechnology Information (NCBI) database. The data can be accessed through the following link: https://dataview.ncbi.nlm.nih.gov/object/PRJNA1203273?reviewer=e5umcfqigkg0md9eqvbb88o3ji. The specific accession number is PRJNA1203273 . The datasets generated during this study are available from the corresponding author upon reasonable request.
